# Why do Individuals Differ in Viral Susceptibility? A Story Told by Model Organisms

**DOI:** 10.3390/v9100284

**Published:** 2017-09-30

**Authors:** Lisa van Sluijs, Gorben P. Pijlman, Jan E. Kammenga

**Affiliations:** 1 Laboratory of Nematology, Wageningen University, 6708 PB Wageningen, The Netherlands; lisa.vansluijs@wur.nl; 2 Laboratory of Virology, Wageningen University, 6708 PB Wageningen, The Netherlands

**Keywords:** model organisms, genetic variation, viral susceptibility, GWAS, QTL, inbred populations

## Abstract

Viral susceptibility and disease progression is determined by host genetic variation that underlies individual differences. Genetic polymorphisms that affect the phenotype upon infection have been well-studied for only a few viruses, such as HIV-1 and Hepatitis C virus. However, even for well-studied viruses the genetic basis of individual susceptibility differences remains elusive. Investigating the effect of causal polymorphisms in humans is complicated, because genetic methods to detect rare or small-effect polymorphisms are limited and genetic manipulation is not possible in human populations. Model organisms have proven a powerful experimental platform to identify and characterize polymorphisms that underlie natural variations in viral susceptibility using quantitative genetic tools. We summarize and compare the genetic tools available in three main model organisms, *Mus musculus*, *Drosophila melanogaster*, and *Caenorhabditis elegans*, and illustrate how these tools can be applied to detect polymorphisms that determine the viral susceptibility. Finally, we analyse how candidate polymorphisms from model organisms can be used to shed light on the underlying mechanism of individual variation. Insights in causal polymorphisms and mechanisms underlying individual differences in viral susceptibility in model organisms likely provide a better understanding in humans.

## 1. Introduction

It is common knowledge that individual people differ in their susceptibilities to different viruses. However, exactly why individuals differ in viral susceptibility is hardly known. Viral susceptibility is a complex phenotypic trait for which there is large variation among individuals regarding infection establishment and development of disease symptoms. The phenotype upon infection is determined by host genes, the environment, and their interactions. Like for many other traits, the genetic architecture is complex, which means that viral susceptibility is associated with multiple genes or loci. Whereas most genes and loci have a small effect on phenotypic traits, few of them have a large phenotypic effect [1,2,3]. Individual phenotypic differences are due to polymorphisms in genes or loci that affect the presence, function, and interaction of host factors, such as RNAs and proteins [4,5].

The detection of polymorphic variants in humans is often based on genome-wide association studies (GWAS) which are currently the most widely used approaches to link genetic variation with viral infection. For instance GWAS detected genetic variants associated with variation in HIV-1, Hepatitis C, dengue, and Influenza A virus infection [6,7,8,9,10,11]. Causal polymorphisms discovered by GWAS are often polymorphic regions that have a large effect on the phenotype. The identification of multiple small-effect polymorphisms is far more challenging due to requirement of large and genetically highly-diverse populations [12]. By definition GWAS correlates genotypic variation with phenotypic variation based on statistical association tests and, as such, GWAS does not provide insight into the underlying molecular mechanisms [13]. Moreover, GWAS is a population-level readout that is difficult to translate to the individual level. Other approaches to find polymorphisms that determine viral susceptibility in humans include specific patients and twin studies. Studies in specific patients typically focus on severe outcomes of disease and can thereby identify large-effect polymorphisms [14,15]. Twin studies are a classical approach to compare the effect of genetics and environment and have identified multiple polymorphisms involved in infectious diseases [16]. However, twin studies also underline the importance of environment, especially as ambient environmental factors can trigger the adaptive immune system to develop further and become more efficient [17]. Both twin and special patient studies require that human subjects are investigated. These may be difficult to find, especially for rare or poorly-studied viral infections.

Model organisms offer alternative opportunities for unravelling the molecular mechanisms that are causal to individual differences in viral susceptibility. Here we review the use of model organisms to study the effect of genetic variation on viral infection. Advanced quantitative genetic tools in model organisms allow for identifying polymorphisms that determine the viral susceptibility in natural populations. The quantitative genetic tools in model organisms provide ways to identify small-effect or rare polymorphisms with an effect on the viral susceptibility. Moreover, in order to mechanistically understand why individuals differ in viral susceptibility, model organisms provide an excellent platform for investigation because individual allelic differences can be studied via experimental manipulation. These fundamental insights can help to guide research in humans through the discovery of homologs or gene networks that underlie natural differences in susceptibility to viral infections.

## 2. Polymorphisms in Host Factors that Interact with Viruses Cause Individual Differences in Viral Susceptibility

**Figure 1:**
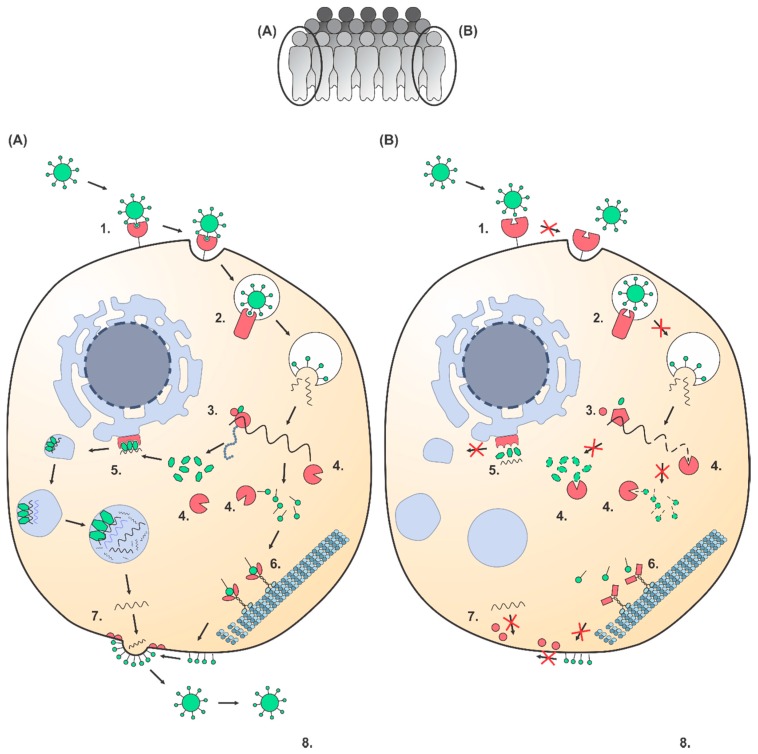
Genetic polymorphisms can affect the viral life cycle in the cell leading to a susceptible and resistant individual – A hypothetical viral life cycle (based on a positive stranded RNA virus) is shown for the cells of a susceptible (**A**) and a resistant (**B**) individual. Host factors are shown in red and viral factors are shown in green. A comparison between the viral life cycles of both cells illustrates several steps where individual polymorphic differences in host factors can affect the viral susceptibility. Step 1: in the susceptible cell the virus binds to the cellular receptor, whereas in the resistant cell the virus cannot enter due to polymorphic changes leading to insufficient binding capacity. *CCR5Δ32* is a well-known polymorphism in a cellular co-receptor preventing HIV-1 entry [19,20,21]. Step 2: in the susceptible cell the virus successfully uses an intracellular transporter, whereas in the resistant cell this is not the case due to genetic individual differences. Polymorphisms in the intracellular receptor *NPC1* can prevent Ebola virus from being released into the host cell [31,32]. Step 3: translation of the viral genome in the susceptible cell is successful, but not in the resistant cell. A polymorphism in a translation initiation factor is associated with resistance to Rice tungro spherical virus [33]. Step 4: host immunity factors recognize the viral genome and proteins in the resistant cell, but natural genetic variation leads to failure to eliminate the virus in the susceptible cell. Multiple viral infections are affected by polymorphisms in the HLA region [12,25,26,27]. Step 5: viral proteins efficiently hijack the cellular machinery for genomic replication, whereas the virus in the resistant cell is unable to replicate due to genetic individual differences. Polymorphisms in the replication gene RPAIN have been associated with Influenza A virus replication [11]. Step 6: viral proteins are transported by the cellular motor proteins in the susceptible, but not in the resistant cell. Step 7: viral egress is facilitated by host factors in the susceptible, but not in the resistant cell. Polymorphisms in *BST2* can prevent HIV-1 from exiting the host cell [22,23,24]. Step 8: the virus is able to infect and replicate in the susceptible individual, in contrast to the resistant individual.

Viruses are obligate intracellular parasites that depend on their host for replication by exploiting various parts of the host cell machinery [18]. At the same time, viruses need to evade or suppress the innate immune system of the host cell to prevent being sensed and eliminated. Viruses interact with host factors, such as cellular receptors and motor proteins, during their life cycle. Proviral host factors are necessary for viral replication, whereas antiviral host factors inhibit or block viral infection. Potentially every polymorphism in a gene encoding a host factor that interacts with a virus may determine individual viral susceptibility (Figure 1). Several polymorphisms in host factors were identified by human population studies and GWAS to affect the viral susceptibility during different stages in the viral life cycle. A polymorphism in the cellular co-receptor CCR5 prevents HIV-1 from entering the cell, making some individuals resistant against HIV-1 [19,20,21]. The polymorphism in the RNA trafficking gene RPAIN is hypothesized to increase viral replication and is associated with severe pneumonia after Influenza A virus infection [11]. The antiviral host factor BST2 restricts viral egress of HIV-1 by tethering the virus to the cell [22] and polymorphisms in BST2 and the regulatory sequences of BST2 are associated with the progression of HIV-1 infection [23,24]. Moreover, several polymorphisms in immune pathways associate with the viral susceptibility of humans. The highly-polymorphic human leukocyte antigen cluster (HLA) regulates the human adaptive immune response. Genetic variation in the HLA underlies susceptibility differences for viral infections, such as HIV-1, Hepatitis B and C virus, Eppstein-Barr virus, and measles virus [12,25,26,27]. Furthermore, polymorphisms in the innate immune sensor MDA5 and in and around the cytokine IFN-λ-3 are associated with Hepatitis C virus clearance and the responsiveness upon IFN treatment [28,29,30].

These examples of polymorphisms detected by GWAS illustrate the power of GWAS to detect genetic variants associated with viral susceptibility. However, GWAS explain a small fraction of the total variation observed, which is in part due to experimental limitations of human GWAS. When a polymorphism is rare and/or has a small-effect on the phenotype, the association will explain only a small part of the total phenotypic variation in the population and is, therefore, not detected by the statistical test [1,34]. Moreover, GWAS in humans have limited possibilities to detect the mechanisms underlying small-effect or rare polymorphisms in the examined population for technical and ethical considerations, e.g., genetic manipulations and experiments cannot be conducted.

## 3. Use of Model Organisms to Unravel the Interplay between Host Genetic Variation and Viral Infection

Quantitative genetic approaches in model organisms provide means to detect genetic variants and the underlying mechanism(s) involved in viral susceptibility [11,35,36,37,38,39,40]. Increased awareness concerning the importance of genetic variation in natural populations has prompted model organism researchers to study the mechanisms of genetic variation using segregating populations generated by parental crossings [5]. The mapping populations consist of genotyped inbred populations, each harbouring different recombinations of the parental alleles. Subsequent phenotyping for viral susceptibility in the inbred strains can yield genetic variants including single nucleotide polymorphisms (SNPs) in coding and non-coding gene regions. Inbred populations with many allelic breakpoints increase the possibility for identification of small-effect or rare polymorphisms because of a high mapping resolution. As many pathways involved in viral infection are conserved across species, the search for genetic variants in model organisms may identify host factors that function similarly to their human homologs.

**Figure 2:**
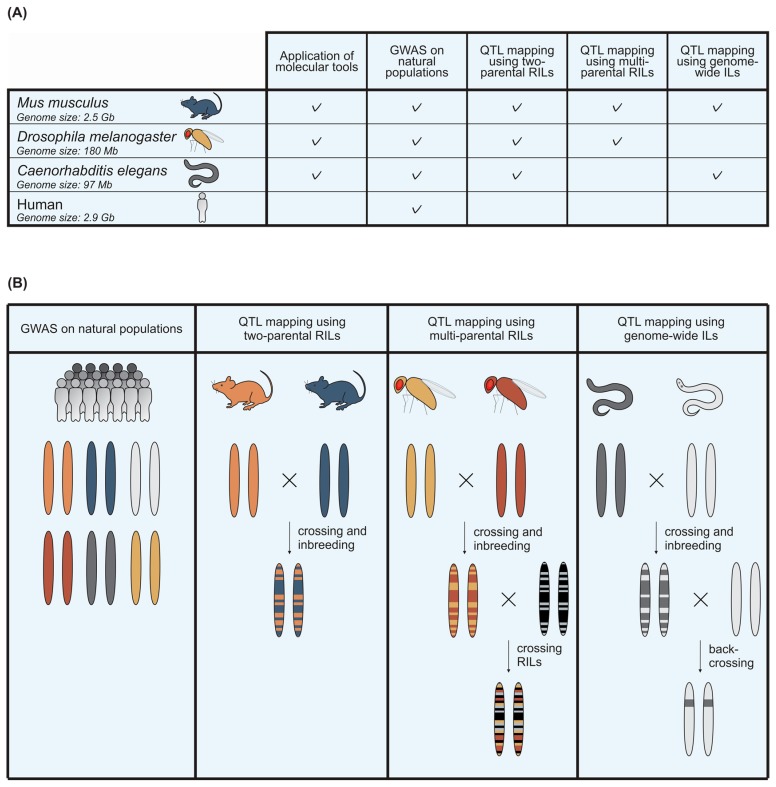
Quantitative genetic tools in model organisms that can be used to study viral infection. (**A**) An overview of the tools that facilitate quantitative genetic studies on viral infection in mice, fruit flies, and nematodes. A comparison is made with the possibilities for human research; (**B**) the genetic composition of several types of quantitative genetic populations. GWAS populations contain individuals with different genetic backgrounds. RIL populations contain the genetic fragments of two strains that are crossed. Multi-parental RIL populations contain genetic fragments from more than two parents, by crossing RILs that originate from distinct parental strains. IL populations contain a single genetic background from one parental strain in the full genome of the other parental strain. ILs are created by backcrossing RILs with one of the parental strains.

Several inbred populations derived from two parents have been created for model organisms which comprise inbred strains of wild isolates, recombinant inbred lines (RILs) and introgression line (IL) populations (Figure 2). RILs and ILs can be used for mapping quantitative trait loci (QTL) associated with viral susceptibility. QTL mapping uses RILs and ILs derived from genetically-divergent parents that differ in susceptibility to virus infection. The parents are crossed and the offspring is inbred to obtain a population of homozygous RILs, each having different genotypes. Once fully genotyped for genetic markers, like SNPs, every individual RIL can be measured for viral susceptibility. QTL mapping statistically correlates viral susceptibility and the genotype of the RILs for every locus on the chromosome. Significant QTL peaks indicate which locus is likely determining the phenotype. In case the detected QTL are relatively broad and cover a large part of the chromosome harbouring many candidate polymorphisms, genetic loci identified in QTL studies can be further fine-mapped with ILs. ILs contain a single genetic fragment (the introgression) of one wild-type strain in the complete genetic background of the other strain. Moreover, a causal relation between the phenotype and the introgression in the target region experimentally verifies the QTL. Next to two-parental RILs, multi-parental RILs can be used to increase the mapping resolution [41,42]. These RILs are created after several rounds of crossing starting with multiple parents, increasing the genetic variation compared to two-parental crosses. A limitation of two-parental RILs is that they do not encompass the full diversity of allelic variation that exists in natural populations. Inclusion of multiple alleles allows for more precise mapping and identification of potential regulatory variants.

Mouse (*Mus musculus*), fruit fly (*Drosophila melanogaster*), and nematode (*Caenorhabditis elegans*) are major model organisms for genetic and molecular research, including virological research focused on pathogenesis, tissue tropism, and (evasion of) immune responses. Below we summarize the quantitative genetic tools that are available for these three model organisms and describe how these tools have been used to identify polymorphisms involved in viral susceptibility (Figure 2). Furthermore, we illustrate how the studies in model organisms can guide detection of genetic variants associated with viral susceptibility in other species, including humans.

## 4. *Mus musculus*

Mice (*Mus musculus*) are widely used model organisms because these small mammals are relatively closely-related to humans [43]. Contrary to other small model invertebrate organisms, in mice the adaptive immune system can be studied. Mice can be infected with several human viruses, such as Influenza A virus and chikungunya virus [44,45]. Moreover, either the mouse (immune system) or the virus can be genetically adapted to facilitate infection with additional human viruses, including Zika and HIV-1 [46,47,48,49,50]. A collection of inbred mice populations is available to investigate genotype-phenotype effects. These populations include the regularly-used multi-parental RILs of the collaborative cross population and the chromosome substitution strains, which can be seen as an IL population with large introgressions [51]. Moreover, RIL and IL populations are also custom-made by researchers to answer specific questions.

Multi-parental RIL mice of the collaborative cross population were infected with a mice-adapted strain of Ebola virus. Some mice strains were completely resistant, whereas others developed the lethal haemorrhagic fever characteristic for Ebola virus infection. Collaborative cross strains with different phenotypes upon infection were crossed after which the viral susceptibility and transcriptional response of the F1 offspring was tested. This approach yielded the identification of two susceptibility loci. One of the loci could be identified in more detail and it was shown that the different susceptibilities to Ebola virus are likely due to distinct *Tie2* (also called *Tek*) polymorphisms [39]. *Tie2* is involved in sepsis upon infection with diverse pathogens and forms a target gene for therapeutics that may relieve Ebola virus infection [52,53].

Chromosome substitution strains contain a chromosome from one parent in the full genome of the other parent; therefore, found QTLs can be specifically attributed to a location [54]. The chromosome substitution strains have been used to study susceptibility differences to the bacterial pathogen *Staphylococcus aureus* and did identify two causal polymorphisms [55]. A similar approach could be taken to study the effect of viral infection in this population.

Experiments combining molecular and quantitative genetic mapping techniques showed that polymorphisms in the gene *Mx1* control several viral infections in mice [38,56,57,58,59,60]. The functioning of *Mx1* against influenza A virus depends on the genetic background of the mice, indicating *Mx1* resistance may be regulated by other, interacting genes [61]. Future studies in mice may show which molecular pathways underlie *Mx1* resistance in different genetic backgrounds. In humans the homolog *MxA* is also a restriction factor of Influenza A virus [62,63], however, phenotypic variation in Influenza A virus susceptibility in humans has not been related to *MxA*. Mice experiments suggest that the genetic architecture underlying *MxA* resistance is complex, therefore, future studies in humans could focus on investigating *MxA* polymorphisms in patients with severe influenza syndromes [64]. A focused search in humans with severe syndromes may identify rare *MxA* polymorphisms, or cases in which the *MxA* polymorphism in combination with the genetic background is deleterious.

Commercially-available and custom-made RILs were used in genetic mapping to reveal a susceptibility locus for West Nile virus in mice. The genetic locus was fine-mapped using custom-made ILs and contains a polymorphism in the gene *Oas1b* (or *2′-5′-OAS1 L1*). *Oas1b* degrades viral RNAs, which explains the differences in West Nile virus susceptibility [37,65]. Subsequently, populations of susceptible humans were analysed to find that polymorphisms in the homolog *OAS1* do indeed affect West Nile virus susceptibility in human [66]. Taken together, these studies illustrate the value of detecting a causal polymorphism in a mouse gene for translational analysis toward detection of causal polymorphisms in human populations.

## 5. *Drosophila melanogaster*

The fruit fly *Drosophila melanogaster* is an important model for studying genetic variation of virus infection, mainly because natural populations can be collected relatively easily which results in the availability of genetically highly-diverse populations. *D. melanogaster* can be infected by at least 30 viruses in nature and around 30% of *D. melanogaster* individuals in the wild carry a viral infection [67]. Researchers using *D. melanogaster* can use the roughly 200 inbred lines of the *D. melanogaster* genetic reference population lines for GWAS [68,69] or the 1700 multi-parental RILs of the Drosophila synthetic population resource for high-resolution QTL mapping [70].

GWAS in the *D. melanogaster* genetic reference population showed that common, large-effect polymorphisms explain most of the phenotypic variation in anti-viral responses against Drosophila Sigma virus and Drosophila C virus [36]. A subsequent QTL mapping using the same viruses in the Drosophila synthetic population resource showed a similar overall trend of large-effect polymorphisms that determine the viral load. However, the QTL mapping technique increased the mapping resolution compared to the previously-performed GWAS; therefore, additional polymorphisms were identified. The additional polymorphisms included one in a rare, but major-effect, gene named *Ge-1* [35]. A polymorphism in *Ge-1* also controls susceptibility towards a rhabdovirus, as identified using a custom-made RIL population. Ge-1 functions as a bridge between two antiviral host factors and the polymorphism disrupts the link between the two binding domains of Ge-1 [71]. These studies illustrate that a rare major-effect gene may be missed by GWAS, but can be identified by QTL mapping.

One of the advantages of *D. melanogaster* is that conclusions based on results obtained in laboratory populations can be investigated in wild populations. GWAS and QTL mapping both found that the *ref(2)P* polymorphism is the major determinant of viral susceptibility in populations in the lab. The function of *ref(2)P* in antiviral immunity links to the innate immunity of the Toll-signalling and autophagy pathways [72]. Field studies confirmed that polymorphisms in *ref(2)P* affect which flies become infected in the wild [73], illustrating the use of *D. melanogaster* to pinpoint polymorphisms that define viral susceptibility in nature. Although the *ref(2)P* polymorphism itself may not hold potential for human therapeutics, studies in fruit flies can clarify how polymorphisms providing resistance spread through natural populations [74], in a similar fashion as these polymorphisms may spread in the human population.

Moreover, *D. melanogaster* can be infected with several human pathogens, such as Sindbis virus and West Nile virus [75,76,77]. These arboviruses are carried by mosquito vectors and both virus and vector can spread quickly due to increased globalisation patterns [78]. Therefore, the diseases that result from the infections are important threats to global health. Genetic mapping in one of the available *Drosophila* panels may unveil polymorphisms that alter the susceptibility of viral vectors and give further insights in the molecular basis of infection.

## 6. *Caenorhabditis elegans*

The self-fertilizing hermaphroditic nematode *Caenorhabditis elegans* has recently become an important model for studying viral genetics. *C. elegans* does not suffer from inbreeding depression, whereas males can be used for genetic exchange [79]. Genetically-diverse wild strains are available, and the overall genetic variation within the species is comparable to humans [80,81]. Existing genetic tools comprise several RIL populations and an IL population that covers the complete genome [82,83,84,85]. ILs can be backcrossed with the parental strain to increase the mapping resolution in target areas [86]. *C. elegans* can be infected with the human zoonotic Vesicular stomatitis Indiana virus and the Orsay virus that is *C. elegans*-specific [87,88,89].

Genetically-diverse wild *C. elegans* strains showed different susceptibilities to the naturally-infecting Orsay virus [40,89,90]. GWAS using a selection of wild strains located a susceptibility locus. Subsequently ILs specific for this location were created by crossing a resistant and a susceptible strain. Experiments in the ILs showed that a *drh-1* polymorphism largely explains differences in viral susceptibility [40]. Mammalian *drh-1* homologs, called RIG-I genes, recognize viruses and trigger the anti-viral response [91]. Therefore, the function of the *drh-1* gene was suggested to be conserved, even though the responding pathways differ [40]. Although this study did not identify a previously unknown gene, studies in *C. elegans* suggest that polymorphisms in *drh-1* homologs may underlie natural differences in viral susceptibility, something that could be investigated in human populations. Moreover, some strains that have the susceptible *drh-1* polymorphism are not susceptible themselves. Follow-up experiments could, therefore, provide additional insights in the role of the genetic background on the functioning of viral sensors like *drh-1*.

## 7. Future Perspectives

Studying viral infection in model organisms provides information that can guide and support research on viral infections in human populations. Current advances, such as the development of advanced multi-parental RILs, improve the mapping resolution and effectiveness of quantitative genetics tools in model organisms. The new mapping tools increase the chances of finding polymorphisms that cause differences in viral susceptibility. Human homologs of causal genes in model organisms are candidate genes that may define viral susceptibility of human populations as well.

The molecular mechanisms behind viral susceptibility differences can be found in vivo in model organisms using transcriptomics, proteomics, and mutational screenings. There is a pleiotropy of techniques available in these models and some recent advances promise to make unravelling molecular mechanisms behind susceptibility differences even more straightforward. Here we highlight only a few promising techniques and suggest how these can be used to address individual differences in viral susceptibility. After identification of a candidate polymorphism homozygous recombination using CRISPR-Cas9 can be applied to change or insert a specific polymorphism in different genetic backgrounds. Therefore, the effect of a specific polymorphism can be tested contrary to other approaches using chemical mutagenesis or knockdown by RNAi. The effect of a polymorphism in a gene may be different than completely knocking-out or knocking-down the same gene. Moreover, advances in transcriptional studies, including RNA-seq or tissue-specific transcriptomics, provide better clues on the pathways involved in the viral susceptibility. Additionally, increasing amounts of big data, including genome sequences and protein structures, can be used to predict the effect of a polymorphism on the functioning of the host factor.

In conclusion, studying the effect of genetic variation on viral infections in model organisms can (a) provide fundamental insights in the molecular and the genetic architecture of viral infection, (b) identify unknown host factors involved in viral infection, and (c) provide candidate genes for human population studies that aim to identify which host factors control individual viral susceptibility.
